# Genotype-Phenotype Associations with Restrictive Cardiomyopathy Induced by Pathogenic Genetic Mutations

**DOI:** 10.31083/j.rcm2306185

**Published:** 2022-05-25

**Authors:** Zhe Yang, Jia Chen, Hong Li, Yubi Lin

**Affiliations:** ^1^The First Dongguan Affiliated Hospital, Guangdong Medical University, 523710 Dongguan, Guangdong, China; ^2^Department of Endocrinology and Metabolism, Zhuhai Hospital Affiliated to Jinan University; The First Hospital Affiliated to Medical College of Macao University of Science and Technology, 519000 Zhuhai, Guangdong, China; ^3^The Second Department of Cardiology, The Second People's Hospital of Guangdong Province, 510310 Guangzhou, Guangdong, China

**Keywords:** restrictive cardiomyopathy, mutations, sarcomeres, phenotype, genotype

## Abstract

Restrictive cardiomyopathy (RCM) is an uncommon cardiac muscle disease characterized by impaired ventricular filling and severe diastolic dysfunction with or without systolic dysfunction. The patients with RCM present poor prognosis and high prevalence of sudden cardiac death, especially in the young. The etiology of RCM may be idiopathic, familial or acquired predispositions from various systemic diseases. The genetic background of familial RCM is often caused by mutations in genes encoding proteins of sarcomeres and a significant minority by mutations in non-sarcomeric proteins and transthyretin proteins. It is important to identify the associations between genotype and phenotype to guide clinical diagnosis and treatment. Here, we have summarized the reported index cases with RCM involving genetic etiology to date and highlighted the most significant phenotype results.

## 1. Introduction

Restrictive cardiomyopathy (RCM) is the least frequently encountered form of cardiac muscle disease, which increases myocardial stiffness and results in impaired ventricular filling [1, 2]. RCM should be classified as either primary or secondary according to underlying etiology [3]. The hallmark of RCM is diastolic dysfunction in the presence of normal or near-normal systolic function, ventricular volumes and wall thickness, at least at the beginning of disease [4]. Consequently, the systolic function might deteriorate at later stages of the disease [5]. Patients with RCM may present signs of left or right heart failure. Right-sided symptoms often predominate, such as peripheral edema and ascites. However, there is a worse prognosis when the left ventricle is affected or ventricular arrhythmias and conduction disturbances are encountered [4, 6]. Pharmacological therapy and heart failure management for RCM show limited efficacy to improve ventricular filling or prolong survival [7]. Although therapy is unsatisfactory, early and accurate diagnosis can significantly improve symptom and survival [4, 7]. The correct diagnosis depends on the distinction between RCM and constrictive pericarditis, which share similar clinical presentations and physical findings [8, 9]. But their pathophysiological mechanism and prognosis differ significantly [10].

RCM may be idiopathic, familial, acquired predispositions from various systemic diseases or a combination of them [2]. Some familial cases presenting genetically determined etiology are often associated with autosomal dominant inheritance or X-linked inheritance [11]. Although familial RCM caused by a single genetic defect is rare in clinical practice, mapping several specific disease-causing genetic mutations resulting in RCM has been recognized [12]. The genetic mutations associated with the occurrence and progression of RCM involve sarcomere proteins, such as troponin I (*TNNI3*), troponin T (*TNNT2*), β-myosin heavy chain (*MYH7*) and α-actin (*ACTC1*) [13, 14, 15]. The patients with RCM owing to sarcomere gene mutations may be accompanied with or without similar microscopic features of hypertrophic cardiomyopathy (HCM) [16, 17]. It used to be considered that RCM and HCM may represent a different phenotype of the same genetic disease [16]. A key piece of evidence was the coexistence of an RCM phenotypic expression with mutations in the HCM-related genes [18]. Other non-sarcomeric gene mutations, including myopalladin (*MYPN*) and titin (*TTN*), and infiltrative RCM-associated mutations have also been identified in RCM recently [11, 19].

This article focuses on heritable genetic mutations and genotype-phenotype associations with familial RCM, as shown in Table 1 (Ref. [11, 13, 14, 17, 19, 20, 21, 22, 23, 24, 25, 26, 27, 28, 29, 30, 31, 32, 33, 34, 35, 36, 37, 38, 39, 40, 41, 42, 43, 44, 45, 46, 47, 48]). The risk stratification and clinical treatment of RCM patients could be affected and improved depending on the systematic databases of genetic alterations.

**Table 1.** **The detailed mutations and their clinical phenotypes associated with RCM**.

Genes	Mutation	Sex	Age	Phenotypes			Complications	Refs
				HCM	DCM	HF		
Sarcomeric Genes								
TNNI3	p.D190H	M	11 y	–	–	+	Marked atrial enlargement	[17]
	p.R192H	M	19 y	+	–	+	Paroxysmal AF, involvement in worst clinical phenotypes	
	p.K178E	F	6 y	–	–	+	Dyspnea, involvement in worst clinical phenotypes	
	p.R145W	M/F	70/68 y	–	–	+	Dyspnea, angina	
	p.A171T	M	63 y	–	–	–	An embolic stroke	
	p.L144Q	F	31 y	–	–	+	–	
TNNI3	p.R204H	F	16 y	+	–	+	–	[20]
TNNI3	p.L144H	F	27 y	–	–	+	Died of pulmonary embolism at 30-year old	[21]
	p.R170Q	M	15 y	–	–	+	Marked atrial enlargement	
TNNI3	p.P150S	M	–	–	–	+	AF	[22]
TNNI3	g.4789_4790delAA	F	6.4 y	–	–	+	–	[23]
TNNI3	g.4762delG	F	23 y	+	–	+	Died of congestive HF	[24]
TNNT2	p.96delE	F	12 m	–	–	+	Involvement in infantile RCM	[25]
TNNT2	p.100-101delNE	F	11 y	+	–	+	Overlap of RCM and HCM phenotypes	[26]
TNNT2	p.I79N	F	53 y	–	–	+	A malignant form of HCM involved	[13]
TNNT2	p.E136K	M	3.5 y	–	–	+	Dysplastic coronaries	[23, 27]
TNNC1	p.A8V and p.D145E	F	8 m	+	–	+	Involvement in young-onset and fatal restrictivephysiology	[28]
MYH7	p.P838L	M	2 m	+	–	+	Early onset, mild hypertrophy, evolution to death quickly	[14]
MYH7	p.G768R	M	15 m	+	–	+	Involvement in restrictive physiology in childhood and HCM in adults	[29]
MYH7	p.R721K	F	–	–	–	+	–	[30]
MYH7	p.Y386C	F	9 m	–	–	+	Myocardial bridging	[31]
MYL3	p.E143K	F	22 y	–	–	+	Severe biatrial enlargement	[32]
MYL2	p.G57E							
TPM1	p.N279H	F	36 y	+	–	+	–	[32]
TPM1	p.E62Q and p.M281T	F	6 y	+	–	+	–	[33]
ACTC	p.D313H	F	8.2 y	–	+	+	A mixed RCM/DCM phenotype	[23]
MYBPC3	p.Q463X	F	34 y	+	+	+	Persistent AF	[34]
	p.E334K	M	45 y	+	+	+	–	
Nonsarcomeric Genes								
DES	p. R16C	M	30 y	–	–	+	AVB, involvement in a recessive phenotype of restrictive physiology	[35]
	p.T453I	M	17 y	–	–	–	AVB	
	p.R406W	M	27 y	–	–	+	AVB, early onset severe cardiac and skeletal myopathy	
	IVS3.del+2_11 TATACCTTGG	F	48 y	–	–	+	AVB	
DES	p.Y122H	M	19 y	–	–	–	AVB	[36]
DES	p.E413K	M	30 y	–	–	+	AVB, severe skeletal myopathy	[37]
DES	c.735G>C	M	41 y	–	–	+	AF, right HF, skeletal myopathy	[38]
MYPN	p.Q529X	M	–	+	–	+	–	[19]
TTN	p.Y7621C	F	35 y	–	–	+	AF, thromboembolism	[11]
FLNC	p.S1624L	F	14 y	–	–	+	Intestinal lymphangiectasia	[39]
	p.I2160F	F	15 y	–	–	+	–	
BAG3	p.P209L	F	15 y	–	–	–	Severe myopathy, neuropathy, long QT syndrome, late-onset RCM	[40, 41]
Infiltrative RCM Pathogenic Mutations								
TTR	p.V122I	–	–	–	–	+	Late-onset RCM, prevalent in African Americans	[42]
	p.I68L	–	–	+	–	+	Male predominance, age-dependent penetrance	[43]
	p.L111M	–	–	–	–	+	Young-onset and manifest RCM, carpal tunnel syndrome	[44]
	p.T60A	–	–	–	–	+	A major determinant of poor prognosis	[45]
	p.H88R	M	65 y	–	–	+	Late-onset cardiomyopathy	[46]
	p.A65G	F	72 y	–	–	+	Hereditary amyloidosis	[47]
	p.S23N	F	46 y	–	–	+	Manifest cardiac and peripheral, ATTR amyloidosis	[48]

M, male; F, female; y, years; m, months; RCM, restricted cardiomyopathy; HCM, hypertrophic cardiomyopathy; DCM, dilated cardiomyopathy; HF, heart failure; AF, atrial fibrillation; AVB, atrioventricular block; –, not mentioned in the previous reports; +, mentioned/occurred in previous reports.

## 2. Sarcomeres and Cardiomyopathies

Cardiac muscle cells (cardiomyocytes), as the structure of myocardium tissue, are composed of parallel bundles of myofibrils with a diameter of about 1 μm. And single myofibril comprises ordered sarcomeres in series, acting as the smallest contractile units of striated muscle [49, 50]. In cardiac muscle cells, sarcomeres, mitochondria, and sarcoplasmic reticulum (SR) account for approximately 60%, 35%, and 5% of the volume, respectively [50].

The sarcomeres are defined as regions residing between the Z-lines (also known as Z-disks or Z-bands) based on the electron-optical properties and structural components [51]. The sarcomeres consist of an A-band flanked by two half I-bands as the central region. The A-band is anisotropic due to parallel aligned thick filaments composed of myosin. The myosin is a hexameric protein including two heavy chains and four light chains. Each myosin molecule contains two myosin “heads”, which are associated with two light chains, respectively, and make a total of four light chains [52]. The myosin “heads” are recognized to reveal the active site for ATP hydrolysis, with which myosin motor proteins produce a force on actin filaments [53]. The I-band on each side of the A-band is nearly isotropic composed of actin and its associated proteins due to thinner and less well-aligned filaments (called thin filaments) [54, 55]. It is well known that the interaction of filamentous actin with myosin is the basis of muscle contraction [56]. The actin, with monomeric (G-actin) polymerized and filamentous (F-actin) states, is the most abundant and highly conserved protein in most eukaryotic cells [57, 58]. G-actin proteins polymerize into long F-actin in the presence of ${\mathrm{Mg}}^{2+}$ and $K^{+}$ at a physiological ionic concentration to form a tight helix. The length stabilization of actin cannot be achieved until the addition of capping proteins to block monomer loss [59]. In addition to the actin backbone, the thin filaments include other two major proteins, tropomyosin (Tm) and troponin (Tn), which are also recognized as significant components regulating the contractile and diastolic system of striated muscle together [60]. Tm is an elongated α-helix molecule that assembles into the parallel dimeric coiled-coil. In response to the binding of a distinct thin filament effector, the Tm moves to a precise location on the actin’s surface to exert its biological activities [61, 62]. Each Tm molecule spans seven actin subunits. A tremendous effort has been made to dissect how Tm proteins transmit the binding event from a single actin monomer to other defined actin monomers according to an accurate activation of actin filaments [63, 64, 65, 66]. The Tn complex consists of three subunits, including Troponin I (inhibitory, TnI), troponin C (calcium-binding, TnC) and troponin T (tropomyosin binding, TnT) proteins [67]. TnI, binding to actin and Tm, functions as an inhibitory subunit to prevent muscle contract without ${\mathrm{Ca}}^{2+}$ binding to TnC, which confers ${\mathrm{Ca}}^{2+}$ sensitivity to the regulatory system. The elongated TnT molecule binds to Tm and interacts between Tm, actin and the rest of the Tn complex, likely modulating the actomyosin ATPase activity [68]. The Tn-Tm complex prevents actin-myosin interactions when the muscle cells are in a state of rest. Conformational changes in the Tn proteins caused by ${\mathrm{Ca}}^{2+}$ released from the sarcoplasmic reticulum enable myosin to bind to actin [51, 69]. These highly ordered sarcomere proteins’ exact structure and relative position are crucial in normal physiological functions, including heart muscle contraction.

The cardiomyocyte cytoskeleton mainly consists of highly ordered sarcomeres referring to myosin-actin and titin filaments (also described as connectin) [70]. The cytoskeleton acts as a sensitive and dynamic cellular organizer and effector rather than a static skeleton responding to extracellular signals. The titin filament, a giant molecular spring and scaffold in cardiomyocytes, spans from the Z-line with NH2 terminus over the half I-band and thick filament to M-line, the centre of sarcomere [71]. Titin protein is potentially expressed in millions of various isoforms of different lengths due to differential splicing within the region of titin located in the I-band from the transcript [72, 73]. Cardiac titin consists of an N2-B segment between the proximal and distal immunoglobulin (Ig) domains and might not match a complementary N2-A component. Therefore, the cardiac titin isoforms are mainly classified as N2-B (3000 kDa in the absence of N2-A) or N2-BA (>3200 kDa at variable sizes in the presence of N2-A) [74, 75]. The expression of titin isoforms in the sarcomere differs according to the species, location and developmental period [76]. The cardiomyocyte compliance is determined by N2-BA/N2-B ratio because the long titin isoform is more compliant than the short one. The normal N2-BA/N2-B expression ratio in the hearts of many adult mammalians, including humans, is approximately 35:65 [77]. There will be less stiffness and resistance to stretching when more N2-BA prevalent [78]. Moreover, oxidation can also affect titin compliance. It had been reported that increased oxidant stress could elevate the stiffness of cardiomyocytes contributing to the global heart stiffening. That is why the aging or failing heart is less compliant [79].

Cardiomyopathies are defined as diseases of the myocardium related to cardiac dysfunction [80], ranging from lifelong symptomless conditions to life-threatening symptoms, including progressive heart failure, different arrhythmias and even sudden cardiac death. Some cardiomyopathies may be idiopathic or familial/genetic/inherited etiology. The genetic studies involving disease-causing mutations suggested that pathological variations in the sarcomere gene played a central role in inherited cardiomyopathies [81]. Several lines of evidence supported that the so-called “disease of the sarcomere” is highly associated with initiation and even different clinical phenotypes of RCM [3]. The observation that the familial occurrence of RCM had long been established firstly attracted attention to its genetic background [82].

## 3. Sarcomeric Gene Mutations

The genetic basis of RCM is largely attributed to mutations in the sarcomeric complex. The main mutations are summarized as follows.

### 3.1 TNNI3 Mutations

TnI has evolved into three isoforms in higher vertebrates, encoded by three related genes: *TNNI1*, *TNNI2* and *TNNI3*. Cardiac TnI (cTnI) in the adult is specifically expressed and regulated by *TNNI3*, slow and fast skeletal muscle cells by *TNNI1* and *TNNI2, *respectively [83]. In the human chromosomal genome, *TNNI3* is located at 19q13.4, encoding approximately 210 amino acids residues with a molecular weight of 24.0 kDa [84]. The functional domain in cTnI between residues from 61 to 112 binds TnT. The inhibitory domain, including residues from 147 to 163, bind strongly to actin and the N-terminal of TnC. It is necessary for regulating the connection of ${\mathrm{Ca}}^{2+}$ to TnC and actomyosin ATPase activity [85, 86]. A second actin-binding site, residues 168 to 188 of cTnI, binds specifically to the actin-tropomyosin filament contributing to the inhibitory activity of cTnI [87]. The C-terminal domain in cTnI is specific and crucial for normal cardiac relaxation. In addition, the remaining C-terminal part residues from 192 to 210 are not fully identified. Still, they are suspected of playing a significant role in stabilizing the ${\mathrm{Ca}}^{2+}$-activated state of tropomyosin in the actin filaments [88].

Most RCM-associated mutations in *TNNI3* are generally missense rather than frameshift or splice mutations. It has been described that a c.87A>G nucleotide substitution in exon 8 of *TNNI3* identified by linkage analysis and direct gene sequencing was highly correlated with marked restrictive filling and a family history of sudden cardiac death [17]. The index case of familial occurrence with RCM involved a proband who suffered from severe heart failure at the age of 11. Subsequent investigation in this study revealed that six missense variants were associated with RCM-related specific genetic mutations: p.D190H, p.R192H, p.K178E, p.R145W, p.A171T and p.L144Q [17]. These mutations largely increase the myofibril sensitivity to ${\mathrm{Ca}}^{2+}$ and affect the basal and maximal actomyosin ATPase activity [89, 90]. The worst clinical phenotypes involved p.K178E and p.R192H resulting in significant increases in ${\mathrm{Ca}}^{2+}$ sensitivity [90]. According to the echocardiography results of a p.R193H transgenic mouse model, there were significantly reduced left ventricular end-diastolic volumes compared with the wild-type group [91]. Moreover, p.R193H mutant of *TNNI3* in adult rat cardiac myocytes further dissected that the increased basal mechanical force cannot be explained by a gain of myofibril ${\mathrm{Ca}}^{2+}$ sensitivity. It was inferred that the TnI-based disinhibition in actin-myosin interaction at normal diastolic ${\mathrm{Ca}}^{2+}$ concentration contributed to the cellular defect of *TNNI3* p.R193H mutation. This ${\mathrm{Ca}}^{2+}$-independent mechanical force was blocked by chronic inhibition of the interaction between actin and myosin proteins [92]. Another heterozygous p.R204H mutation in exon 8 of *TNNI3* was identified in a young female patient with pure RCM who had undergone heart transplantation at the age of 23 [20]. The specific mechanism of how p.R204H mutation induces primary RCM is still unclear, although the phenotype and clinical condition deteriorated rapidly. Additionally, two novel disease-causing p.L144H and p.R170Q missense mutations, both present in exon 7 of *TNNI3*, were found in a family with four affected patients and a single unrelated patient essentially associated with RCM [21]. The p.L144H mutation was located in the first actin-binding domain and overlapped with the ATPase inhibitory domain. The p.R170Q mutation was located in the second actin-binding domain [93]. These mutations within the actin-binding domain have been presented to cause excessive inhibition in troponin I actomyosin ATPase activity [86]. Some studies at the laboratory suggest that these mutations could weaken the ability of the troponin complex to sufficiently inhibit the cross-bridge attachment when muscle cells are at the relaxation phase, which significantly decreased the rate of muscle relaxation [94, 95]. The change costs higher energy to return to the pre-contractile basal state [21]. Furthermore, a pathogenic p.P150S in exon 7 of *TNNI3* responsible for RCM was confirmed in a Chinese family [22]. This mutation is in the actin and the N-terminal of the TnC binding domain where the configuration of cTnC and cTnI returns to the status before constriction and cTnI binds to actin again as a consequence of decreased ${\mathrm{Ca}}^{2+}$ concentration. However, the process fails to complete once mutation occurs in this domain, such as p.P150S, resulting in impaired diastolic filling in patients with RCM.

Besides missense mutations, deletion mutations in *TNNI3* are also responsible for the development of RCM in a tiny percentage of patients. A novel deletion mutation of two nucleotides g.4789_4790delAA in exon 7 of *TNNI3* was identified in RCM individual [23]. This mutation contributed to a frameshift and the presence of a premature termination codon at amino acid site 209 (E177fsX209). Another index patient diagnosed with RCM at the age of 23 and died due to progression of congestive heart failure at the age of 28 indicated a deletion of one nucleotide g.4762delG in exon 7 of *TNNI3*. This deletion also induced a frameshift in residue 168 and the introduction of a premature termination codon at site 176 (D168fsX176) [24]. According to laboratory tests, this mutation resulted in the truncation of the C-terminal part of cTnI and an approximate 50% decrease in total cTnI, likely leading to a nearly total deficiency of the second actin TnC binding domain. The damage of the inhibitory effect of the Tn-Tm complex on thin filaments could cause impaired myocardium relaxation and restrictive filling [24].

Collectively, the integrity of the cTnI is essential for conformation of the Tn complex in myofilament and the inhibition of actomyosin ATPase activity. To dissect the pathogenic cellular mechanisms resulting from *TNNI3* mutations to identify the cause of RCM is scientifically and clinically important.

### 3.2 TNNT2 Mutations

*TNNT2* gene encodes the Tm-binding subunit of the Tn complex in the heart, which acts as a regulator of striated muscle contraction in response to differential intracellular ${\mathrm{Ca}}^{2+}$ concentration [96]. It is well established that the association between pathogenic *TNNT2* mutations and risk of cardiomyopathies [25].

The first case of RCM caused by a *de novo* mutation of *TNNT2* was reported in a 12-month-old girl [97]. This infantile case had experienced recurrent episodes of sinus bradycardia and tachycardia, malignant ventricular arrhythmias and hemodynamic instability. She received extracorporeal membrane oxygenation therapy, followed by a biventricular assist device insertion and subsequently underwent heart transplantation [97]. Genetic testing revealed a novel deletion mutation c.285_287GGA in exon 9 of *TNNT2*, resulting in deletion of glutamine in 96 amino acid residual (p.96delE). The p.96delE mutation is located in the highly conserved domain. It induces the deficiency of a negative charge in the coiled-coil region, affecting the TnT-Tm-actin complex’s interactions [98]. Following experimental results demonstrated that p.96delE mutation significantly increased the ${\mathrm{Ca}}^{2+}$ sensitivity in fibres reconstituted with the adult and fetal TnT isoforms. However, the effect was enhanced in adult Tn protein [99]. Another heterozygous in-frame double deletion mutation (c.297-302AATGAG) in exon 9 of *TNNT2* was reported in an RCM pediatric patient. That led to the deletion of asparagine and glutamic acid, two highly conserved amino acids, at positions 100 and 101, respectively (p.100-101delNE) [26]. This case’s clinical condition deteriorated rapidly with frequent chest pain and dyspnea, and the patient ultimately received a heart transplant 15 months after initial presentations. Histology indicated mild muscle hypertrophy, interstitial fibrosis and disarray of the myocytes. It must be mentioned that those observations revealed a certain overlap of restrictive and hypertrophic phenotypes that coexisted in this RCM case.

Some missense mutations proved to be associated with RCM. In a large family with autosomal dominant cardiomyopathy, the c.236T>A missense mutation in exon 8 of *TNNT2* led to the substitution of isoleucine (I) with asparagine (N) at amino acid position 79 (p.I79N) [13]. RCM caused by this mutation often complicated massive biatrial enlargement, markedly abnormal diastolic function, subsequent sinus bradycardia and progression to complete heart block, and even needed radiofrequency ablation and pacemaker/cardioverter-defibrillator implantation therapy in some patients. A transgenic mice model with targeted human cTnT (*TNNT2 *p.I79N) protein expression showed enhanced calcium-activated force generation and ATPase activity without muscle hypertrophy. The rate of ${\mathrm{Ca}}^{2+}$ dissociation from TnC during diastole decreases, and the baseline muscle tension increases, resulting in slower relaxation, the elevation of end-diastolic pressure and subsequent diastolic heart failure [100, 101]. Another index RCM case induced by a novel nucleotide substitution g.9718G>A in exon 10 of *TNNT2* was associated with myocyte vacuolation according to the histology of proband’s explanted heart. This disorder is commonly observed in TnT-mutation related cardiomyopathy [23, 27]. The underlying pathogenicity of this new variant remains to be elucidated.

Therefore, the identified *TNNT2* mutations, such as p.100-101delNE and p.I79N, associated with RCM often occur in a TnT binding fragment corresponding to residues 70–170 in the N-terminal domain. This finding suggests the existence of a mutational hotspot region in *TNNT2* where mutations may result in impaired Tm-dependent functions of cTnT [102].

### 3.3 TNNC1 Mutations

Cardiac troponin C (cTnC) consists of two globular EF-hand (the most common calcium-binding motif) domains and a flexible linker. The calcium-sensing part of the Tn complex is troponin C encoded by *TNNC1* in both cardiac muscle and slow skeletal muscle. There are two high-affinity calcium-binding sites in the C-domain of cTnC where are often occupied by ${\mathrm{Ca}}^{2+}$ in physiologic conditions [103].

Previously, mutations in* TNNC1* have been associated with HCM or DCM. Nowadays, the evidence indicated that a compound heterozygous mutation p.A8V (c.C23T) and p.D145E (c.C435A) in *TNNC1* inducing fatal RCM was described in a pediatric proband who inherited the mutation from her unaffected paternal grandmother and maternal grandfather, respectively [28]. The younger sister of this proband, who carried the same genetic background, initially showed congenital HCM, evolved to RCM, subsequently occurred with heart failure and death. This phenomenon suggests that RCM induced by the compound heterozygosity p.A8V and p.D145E is combined with a young-onset marked restrictive physiology, familial history of sudden cardiac death and gradually evolves into septal hypertrophy. The p.A8V mutation alone caused a more open cTnC N-domain conformation, presumably increasing interactions with the switch region of cTnI [104], while the p.D145E mutation altered ${\mathrm{Ca}}^{2+}$ bind by the C-domain of cTnC [105]. It appeared not compatible with the fact that the grandparents of the proband who carried single p.A8V or p.D145E allele were unaffected. The seemingly contrasting finding might be explained by the possibility that the single mutation was haploinsufficiency to cause a complete penetrance. However, the combination of compound heterozygotes p.A8V and p.D145E resulted in a more severe phenotype of RCM. Experimental results demonstrated that the major abnormality induced by p.A8V and p.D145E mutations at the same time was the decreased ${\mathrm{Ca}}^{2+}$ off-rate, altered muscle relaxation and impairing diastolic function [106, 107].

### 3.4 Myosin Associated Mutations 

As described above, myosin is a hexameric contractile protein containing two heavy chains (MHC, encoded by *MYH7* in the heart) associated with four light chains (MLC). The four MLCs are classified as two regulatory light chains (encoded by *MYL2* in the heart) and two essential light chains (encoded by *MYL3* in the heart). The C-terminal part of each MHC is α helical, whereas its N-terminal part folds into a globular head region called subfragment 1 (S1). The S1 contains a motor domain binding to actin. It hydrolyses ATP and a neck domain composed of a regulatory and essential light chain, respectively, functioning as a lever for filament sliding in contraction [108, 109].

A *de novo* heterozygous mutation p.P838L was firstly identified in *MYH7* in an infantile RCM case. The clinical presentation of this proband was characterized by early-onset, mild hypertrophy of the left ventricle and a very short evolution to death [14]. The p.P838L mutation is located in an extremely conserved hinge segment between the rod region and the globular head region of myosin protein. The marked restrictive physiology might result from the myosin head region’s impaired flexion during the relaxation cycle. However, in a p.P838L myosin transgenic Drosophila melanogaster model, the heart morphology and cardiac function was normal, although the p.P838L mutant myosin increased basal ATPase, actin sliding velocity, rotational flexibility and the average angle of two heads *in vitro * [110]. On the one hand, the seemingly different findings might result from the possibility that Drosophila myosin protein is less sensitive to the p.P838L perturbation than humans. On the other hand, the identification of the human pathogenic mutations involved sequencing of select candidate genes. Hence, it is possible that a mutation in another genetic locus, alone or in conjunction with P838L myosin, is responsible for the severe phenotype observed in the human patients [110]. Another missense mutation, p.G768R in exon 21 of *MYH7*, also was found in a pediatric RCM case [29]. The p.G768R locates in a highly conserved region across species and has previously been reported as a disease-causing mutation associated in adults with HCM [111]. It suggests that the phenotypic manifestations of *MYH7* mutations in children, especially young ones, are different from adult ones. Further investigations are needed to determine whether other untested genetic mutations or sensitive indicators functioned as potential contributors to the severity and age of onset. A novel *MYH7* p.R721K mutation was found in an RCM proband, who died at 47-year old due to progressive congestive heart failure, and her young son both showed biatrial enlargement, normal wall thickness and restrictive features. Yet, her other non-carrier son did not have these features [30]. The p.R721K mutation located in the converter domain of *MYH7* affects myosin’s ATPase activity. RCM induced by *MYH7* mutation in this domain is associated with severe diastolic heart failure, high rates of atrial fibrillation, stroke, poor prognosis and even sudden cardiac death [30]. Another p.Y386C mutation was reported in exon 13 of *MYH7*, which was previously seen in an infant with *de novo* HCM by the laboratory. The index case died at the age of 18 months, and the autopsy findings presented RCM, not HCM [31]. Interestingly, this is the first observation in a patient with RCM overlapped myocardial bridging under an *MYH7* mutant background.

RCM caused by MLC-related mutation was firstly reported in an El-Salvadoran 22-year-old female. The patient underwent recurrent syncope and severe heart failure [32]. There were homozygous mutations of *MYL3* p.E143K (c.427G>A), combined with a novel heterozygous mutation of *MYL2* p.G57E (c.170G>A). Her mother, who carried a double heterozygous *MYL3 *p.E143K and *MYL2 *p.G57E, showed a normal echocardiogram and electrocardiogram examinations. According to this phenomenon, the homozygous *MYL3 *p.E143K was highly considered to contribute to RCM in the proband [32].

### 3.5 TPM1 Mutations 

The α-tropomyosin is encoded by *TPM1* and plays a crucial role in actin regulation and stability, participating in fundamental functions in heart development. Mutations in *TPM1* cause dominantly inherited cardiomyopathies [112]. Almost all recently reported *TPM1* variants are missense mutations that resulted in a single amino acid substitution.

A novel homozygous missense mutation *TPM1 *p.N279H (c.835A>C) was found in an Italian RCM case. The endomyocardial biopsy showed mild myocyte hypertrophy and no evidence of amyloid or iron deposition [32]. This proband’s father carried heterozygous p.N279H mutation and was diagnosed with HCM in the absence of restrictive physiology. In 2021, the compound heterozygous *TPM1* variants p.E62Q (c.184G>C) and p.M281T (c.842T>C) were identified in a child with RCM for the first time [33]. This proband was diagnosed with RCM at the age of 6, received orthotopic heart transplantation at 12-year old, and reached adult age without cardiovascular events. In addition, the family members of the proband carrying one of these two mutations presented HCM phenotypes. Following tests suggested that *TPM1* mutations resulted in time-dependent and progressive deterioration of cardiomyocyte CaT amplitudes. Yet, the reduced CaT amplitudes and the deficient sarcomeric structures are independent of the *TPM1* mutations and the clinical phenotypes of cardiomyopathies [33].

### 3.6 ACTC1 and MYBPC3 Mutations 

Actin is a highly conserved protein and encoded by* ACTC1*. Mutations in this gene have been phenotypically associated with various cardiac abnormalities. A novel p.D313H mutation (g.4642G>C) in exon 5 of *ACTC1* was observed in an individual with RCM [23]. Interestingly, the proband’s father died from DCM after heart transplantation and the older sister was diagnosed with overlapping phenotypes of RCM and DCM. The p.D313H was located in the immobilized region of the actin filament, acting as an important tropomyosin-binding site [113]. The specific mechanism by how the p.D313H mutation induced various clinical phenotypes of cardiomyopathy remains unclear.

The *MYBPC3* gene encodes the cardiac isoform of myosin-binding protein C (MyBPC), a myosin-associated and large multi-domain protein. The role of MyBPC in the sarcomere regulation is not yet fully understood. Previously, *MYBPC3* mutations were demonstrated highly related to familial HCM [114]. However, a nonsense mutation *MYBPC3* p.Q463X (c.1387C>T) was identified in a multigenerational family with three adult RCM patients. Moreover, another missense mutation *MYBPC3* p.E334K (c.1000G>A) was observed in an unrelated patient [34]. A zebrafish model with genetic knockdown of *MYBPC3* showed ventricular hypertrophy and diastolic heart failure manifestations, including decreased diastolic relaxation velocity, pericardial effusion and dilatation of the atrium [115]. It is noted that primary RCM caused by *MYBPC3 *mutation is associated with severe diastolic dysfunction, yet the long-term prognosis is still obscure [34].

## 4. Nonsarcomeric Gene Mutations 

While nonsarcomeric mutations-associated RCM subtypes are less common, several mutations have recently been identified in index cases.

### 4.1 DES Mutations 

Desmin is encoded by *DES* and functions as the chief intermediate filament of the skeletal and cardiac tissue connecting the Z-bands to the subsarcolemmal cytoskeleton. Cardiomyopathies caused by *DES* mutations often present severe restrictive physiology, syncope, sudden cardiac death due to conduction defect and overlap with a heterogeneous group of skeletal myopathies [116].

In four unrelated probands with RCM complicated with the atrioventricular block (AVB), there were three novel mutations p. R16C, p.T453I, a 10-bp deletion at the exon-intron boundary of exon 3 and one known heterozygous mutation p.R406W identified in *DES * [35]. The novel p.R16C mutation was associated with a recessive phenotype due to the absence of RCM in three heterozygous carriers. The novel p.T453I mutation is located in the highly conserved 9-amino acid motif among type III intermediate filaments acting as desmin interaction with other cytoskeletal proteins [117]. The new 10-bp deletion at the exon-intron boundary of exon 3 damaged the exon 3 donor splice site, predicting the loss of 32 amino acids and the accumulation of desmin-positive material [118]. The known p.R406W mutation was located in the C-terminal of the desmin core domain and associated with early-onset severe cardiac and skeletal myopathy [119]. Although the probands carry different mutations affecting different domains, all shared the identical cardiac phenotypes of RCM in combination with AVB [35]. Another novel homozygous missense mutation *DES* p.Y122H (c.364T>C) was also reported in the index patient with RCM plus AVB [36]. Following experimental results *in vitro* revealed a severe filament assembly defect of mutant DES protein. The novel *DES* p.E413K mutation was identified in a family with pure RCM, including three affected and five at-risk members. The pathogenicity of p.E413K mutation at a highly conserved end of the alpha-helical rod domain might induce potential disruption of intramolecular interactions and inability of filamentous cellular network [37]. Recently, in an index patient with RCM in combination with atrial fibrillation, there was a heterozygous mutation c.735G>C in *DES*. This mutation affected the last base pair of exon 3 and caused a splice site defect. RCM caused by this mutation showed right heart failure, massive dilation of the right atrium and recurrent atrial fibrillation [38].

### 4.2 MYPN Mutations

The *MYPN* gene encodes myopalladin protein connecting structural regulatory molecules by translocation from the Z-lines and I-bands to the cardiomyocyte nucleus [120]. Mutations in *MYPN* associated with DCM, HCM and RCM have been reported. *MYPN* p.Q529X mutation was identified in siblings with RCM, yet their carriers’ mother was not affected. Different phenotypes were observed in family members carrying the same mutation [19]. The phenomenon of reduced penetrance of p.Q529X mutation might be explained by the possibility that the patients with RCM had other disease-associated mutations inherited from their father and absent from their mother. The knock-in heterozygote *MYPN* p.Q526X mutant mice revealed the diastolic dysfunction and restrictive physiology. There was preserved systolic function without overt hypertrophic remodeling. The phenotype of mutant mice resembles RCM induced by p.Q529X in humans [121].

### 4.3 TTN Mutations

*TTN *(also called* titin*) gene encodes the largest human protein consisting of 364 exons and approximately 38,000 amino acid residues with a molecular weight of 4200 kDa. The TTN protein provides architectural support and sarcomeric organization during muscle contraction [122]. Mutations in *TTN* refer to the different phenotypes of cardiomyopathies and missense variants are very common and frequently benign in DCM [123]. A linkage analysis study identified a missense mutation* TTN* p.Y7621C (c.22862A>G) in a family with RCM involving six affected individuals aged 12–35 years [11]. The p.Y7621C mutation is located in titin’s most highly conserved A/I junction region, connecting the compliant I-band and the rigid thick filament bound A-band. The clinical presentations of RCM induced by *TTN* mutation showed severe diastolic dysfunction overlapped with atrial fibrillation and thromboembolic phenomena.

### 4.4 FLNC and BAG3 Mutations

Filamin C is an actin-cross-linking protein encoded by *FLNC* in heart muscle. Pathogenic mutations in *FLNC* have been reported to cause dominant isolated cardiomyopathy phenotype. The prevalence of patients carrying a unique *FLNC* pathogenic mutation in a cohort was evaluated 8% in RCM [124]. It has been identified that two novel missense mutations, *FLNC* p.S1624L and p.I2160F, were associated with familial RCM. It was suspected that *FLNC* and *DES* mutations shared similar pathological mechanisms due to identical behaviour of cytoplasmic aggregation [39].

Mutation in *BAG3* is a rare cause of RCM. Recently, a heterozygous mutation p.P209L (c.626C>T) in exon 3 of *BAG3* was found in a 15-year-old girl. The proband showed severe myopathy, neuropathy, asymptomatic long QT syndrome and late-onset RCM [40]. The *BAG3* p.P209L mutation was also present in another index patient who suffered from severe myofibrillar myopathy and RCM [41].

## 5. Infiltrative RCM-Associated Mutations

Cardiac amyloidosis (CA) is considered as the prototype of the infiltrative form of RCM. Although CA can be acquired, there are several mutations in genes involving transthyretin (TTR). TTR primarily serves as a transporter for thyroxin and for retinol-binding protein. This protein is a tetramer, but has an innate ability to dissociate into monomers which tend to be amyloidogenic properties. There are three main types of CA: immunoglobulin light chain cardiac amyloidosis (AL-CA), wild-type transthyretin cardiac amyloidosis (ATTRwt-CA) and mutant transthyretin cardiac amyloidosis (ATTRm-CA) [3].

ATTRm-CA is an autosomal-dominant disease in which gene mutations lead to changes in the protein TTR. The clinical symptoms vary extensively depending on many factors including specific *TTR* mutation site and geographical distribution. The *TTR* p.V30M mutation was the most common worldwide which induces progressive peripheral sensory-motor polyneuropathy with later cardiac manifestations. However, other mutations p.V122I, p.I68L, p.L111M and p.T60A cause exclusively infiltrative cardiomyopathy [125]. The p.V122I mutation is prevalent in 3.4% of African Americans, and the clinical phenotype often refers to late-onset RCM, despite a low clinical penetrance of the disease [42]. Another prospective observational Atherosclerosis Risk in Communities study reported that the p.V122I carriers had an increased risk of heart failure during the later years compared with non-carriers, indicating that p.V122I carriers are predominantly at increased risk of heart failure with an age-dependent penetrance [126, 127]. The p.I68L mutation is endemic in central-northern Italy and presents as HCM or RCM. Male preponderance is present in affected patients but not in unaffected mutation carriers [43]. The cardiac mutation p.L111M has been traced to three unrelated Danish families [44]. The patients showed developing or manifest RCM with a diastolic dysfunction as the first sign of disease. Ischemic symptoms were often present in the form of angina pectoris because of amyloid deposits in the coronary arteries [44]. Significantly, these patients with the p.L111M were younger and less likely to be male [128]. Familial amyloid polyneuropathy (FAP) resulting from the *TTR *p.T60A mutation was firstly described in 1986 in an Irish family [45]. Moreover, cardiac amyloidosis is always present at diagnosis in FAP p.T60A mutation, and is a major determinant of its poor prognosis. Other sporadic cases of RCM associated with *TTR *mutations such as p.A65G, p.H88R and p.S23N have recently been reported [46, 47, 48].

## 6. Conclusions and Perspectives

Previously, the invasive endomyocardial biopsy was needed to diagnose the primary cardiomyopathy, and genetic testing was probably underestimated. Nowadays, identifying disease-causing mutations in cardiomyopathy has shed new light on molecular mechanisms. Given the ever-broadening link between specific phenotype of RCM and pathogenic mutations, genetic testing would be advantageous to patients with severe diastolic dysfunction. Location of the mutation in gene influences the development of clinical phenotype of RCM. Therefore, recognizing the effects of shared genetic mutations and establishing a close association with clinical phenotypes is a major aim of future studies.

## Data Availability

The data used in this study is not publicly available, but it might be available from the corresponding author upon reasonable request and permission from relevant Chinese Authorities.

## Abbreviations

RCM, restrictive cardiomyopathy; HCM, hypertrophic cardiomyopathy; DCM, dilated cardiomyopathy; *TNNI3*, troponin I; *TNNT2*, troponin T; *MYH7*, β-myosin heavy chain; *ACTC*, α-actin; *MYPN*, myopalladin; *TTN*, *titin*; SR, sarcoplasmic reticulum; Tm, tropomyosin; Tn, troponin; MHC, myosin heavy chain; MLC, myosin light chain; MyBPC, myosin-binding protein C; AVB, atrioventricular block; TTR, transthyretin; CA, cardiac amyloidosis; AL-CA, immunoglobulin light chain cardiac amyloidosis; ATTRwt-CA, wild-type transthyretin cardiac amyloidosis; ATTRm-CA, mutant transthyretin cardiac amyloidosis; FAP, familial amyloid polyneuropathy.
